# Within-Gender Changes in HIV Prevalence among Adults between 2005/6 and 2010/11 in Zimbabwe

**DOI:** 10.1371/journal.pone.0129611

**Published:** 2015-07-24

**Authors:** Elizabeth Gonese, Tonderai Mapako, Janet Dzangare, Simbarashe Rusakaniko, Peter H. Kilmarx, Maarten J. Postma, Stella Ngwende, John Mandisarisa, Ponesai Nyika, David A. Mvere, Owen Mugurungi, Mufuta Tshimanga, Marinus van Hulst

**Affiliations:** 1 U.S. Centers for Disease Control and Prevention (CDC), Harare Zimbabwe; 2 National Blood Service Zimbabwe, Harare Zimbabwe; 3 Unit of Pharmaco-Epidemiology & Pharmaco-Economics (PE2), Department of Pharmacy, University of Groningen, Groningen, Netherlands; 4 Ministry of Health and Child Welfare, Harare, Zimbabwe; 5 Department of Community Medicine, University of Zimbabwe, Harare, Zimbabwe; 6 Division of Global HIV/AIDS, CDC, Atlanta, United States of America; 7 National Microbiology Reference Laboratory, Harare, Zimbabwe; 8 Department of Clinical Pharmacy and Toxicology, Martini Hospital, Groningen, Netherlands; UCL Institute of Child Health, University College London, UNITED KINGDOM

## Abstract

**Introduction:**

Zimbabwe has reported significant declines in HIV prevalence between 2005/06 and 2010/11 Demography and Health Surveys; a within-gender analysis to identify the magnitude and factors associated with this change, which can be masked, is critical for targeting interventions.

**Methods:**

We analyzed change in HIV prevalence for 6,947 women and 5,848 men in the 2005/06 survey and 7,313 women and 6,250 men in 2010/11 surveys using 2005/06 as referent. The data was analyzed taking into consideration the survey design and therefore the svy, mean command in *Stata was used* in both linear and logistic regression.

**Results:**

There were similar proportional declines in prevalence at national level for males (15% p=0.011) and females (16%,p=0.008). However, there were variations in decline by provincial setting, demographic variables of age, educational level and some sexual risk behaviours. In logistic regression analysis, statistically significant declines were observed among men in Manicaland, Mashonaland East and Harare (p<0.01) and for women in Manicaland, Mashonaland Central and Harare (p<0.01). Although not statistically significant, numerical increases were observed among men in Matebeleland North, Matebeleland South, Midlands and for both men and women in Bulawayo. Young women in the age range 15-34 experienced a decline in prevalence (p<0.01) while older men 30-44 had a statistically significant decline (p<0.01). Having a secondary and above education, regardless of employment status for both men and women recorded a significant decline. For sexual risk behaviours, currently in union for men and women and not in union for women there was a significant decline in prevalence.

**Conclusion:**

Zimbabwe has reported a significant decline among both men and women but there are important differentials across provinces, demographic characteristics and sexual risk behaviours that suggest that the epidemic in Zimbabwe is heterogeneous and therefore interventions must be targeted in order to achieve epidemic control.

## Introduction

Sub-Saharan Africa is experiencing an HIV epidemic with substantial variation across and within countries.[1] The Joint United Nations Program on HIV/AIDS (UNAIDS) reported that among the worst affected countries, Ethiopia, South Africa, Zambia and Zimbabwe had reduced new HIV infections by more than 25% between 2001 and 2009.[1] National HIV prevalence declined in Kenya, stabilized in Uganda, Rwanda and Nigeria.[1] However HIV prevalence increased in Mozambique and remained high in Swaziland and Botswana.[1]

As countries battle to curb and reverse the impact of the HIV epidemic, understanding the influence of demographic, behavioral and biological determinants of the HIV epidemic remains key to evidence based programming and policy formulation. Evidence from the analysis of antenatal clinic surveillance (ANC) and Demographic and Health Survey (DHS) data has shown that age, sex, educational level and residence are important differentials for HIV epidemics in Sub-Saharan Africa. Heterosexual transmission remains the main driver of the HIV epidemic in sub-Saharan Africa. Women are disproportionately affected by the epidemic as a result of the biological factors and cultural socio-economic disparities [2,3]

Analysis of data in Zambia, showed significant declines in HIV prevalence among women aged 15–19 years attending sentinel antenatal clinics, whereas data from DHS showed that HIV prevalence declined in urban women aged 15–29 years and in the 15–24 year group among rural women.[4] A follow-up analysis by Kayeyi et al, comparing the 2001/2 and 2007 DHS and 1994 to 2008 ANC data concluded that national level trend analysis masks important differences in the change in HIV prevalence by geographic setting and level of educational attained. [5]

Analysis of eight national demography and health surveys conducted between 2003–2005 in Sub Saharan countries (Kenya, Ghana, Burkina Faso, Cameroon, Tanzania, Lesotho, Malawi, and Uganda) showed a positive association of wealth and HIV prevalence. This association was partially explained by other factors such as such as place of residence, education, and by differences in sexual behaviour, such as multiple sex partners, condom use, and male circumcision.[6]

An extended analysis of the two DHS conducted in 2003/4 and 2007/8 in Tanzania showed that age at first marriage, cohabiting, multiple sexual partners and presence of sexually transmitted infections (STI) were associated with HIV infection among women. Further, the analysis showed that while overall HIV prevalence decreased by 14% (7.9% to 6.8%), this decline was only significant among urban males; prevalence did not decline significantly in other socio-demographic, behavioral and biological sub-groupings. This and other analyses highlight the benefit of disaggregated analysis of data. [7–10]

Zimbabwe has one of the highest HIV burdens in Southern Africa with a generalized epidemic in which an estimated 860,000 adults (15–49 years) living with HIV.[11]Antenatal sentinel surveillance has reported a decline from 25.7% in 2002, 21.3% in 2004, and 17.7% in 2006 to16.1% in 2009.[12] Because of the limitations of ANC data in representing the general population, Zimbabwe implemented its first DHS with HIV testing in the general population in 2005/06 and a follow-up in 2010/11. These surveys showed a decline in HIV prevalence in Zimbabwe from 18.1% (95% CI 16.9–19.3) (2005/2006) to 15.2% (95% CI 14.3–16.1)(2010/2011). [13,14] The national trend showed a decrease in HIV prevalence of 15% in men and 16% in women between the 2005/6 and 2010 DHS. Because of the growing evidence that national trends tend to mask important differences, we set out to examine the within gender differentials in the change in HIV prevalence. This analysis was focused to identify the key factors influencing this change in prevalence and so we examined differences by geographic location, selected demographic, behavioural and biological characteristics. Results of such an analysis are useful in targeting interventions.

## Methodology

### Study Design

This is a descriptive cross-sectional study focusing on the within gender changes of HIV prevalence between the DHS 2005–6 and 2010/11.

### Study Setting

Zimbabwe is a landlocked, bordered by Mozambique on the east, South Africa on the south, Botswana on the west, and Zambia on the north. Based on Census of 2012 the total population of the country was 13,061,239. There were 6,280,539 males and 6,780,700 females giving a sex ratio of 93 males per 100 females. [15] The population was relatively young with 41 percent of the population being below age 15 years and about 4 percent age 65 years and above.

### Population and Sampling

The study population consists of 6,947 women and 5,848 men in 2005/6 and 7,313 women and 6,250 men in 2010/11 in the age group 15–49 that were eligible (de facto that is those who stayed in the household last night), completed the interviews and consented to HIV testing in the respective DHS. All the HIV data from these DHS was included in our analysis and we did not do further sampling over and above what was done in the original reports of the ZDHS 2005/6 and 2010/11. Therefore the data collection process and sampling procedures in our study are the same as those for standard DHS methods described in main reports ^9,10^. In summary, in both DHSs a two stage sampling method was used to select households for participation in survey. The 2002 Zimbabwe Population Census used the national administrative subdivision of provinces and districts to further create wards and enumeration areas (EA). The 2005/06 and 2010/11 surveys selected a representative sample of EA from which households were selected in the second stage of sampling.

### HIV Testing

Consenting individual participants were interviewed and provided a blood sample from which a dried blood spot was prepared. Enzyme-linked immuno assays were used to test the dried blood spots samples for HIV at the National Microbiology Reference (NMRL). All samples were tested with first ELISA assay test. All positives were subjected to a second ELISA. If the test result was positive on both tests then the final result was tendered positive, discordant samples were retested with a Western Blot to confirm whether the result was negative or positive. If the result was still discordant, the sample was rendered indeterminate. The HIV results were used to create a laboratory dataset which were delinked from the individual questionnaire dataset.

### Variables

The explanatory variables selected for use in our study are for (a) socio-demographic factors that include province (ten administrative provinces), location (urban, rural) as defined by the Zimbabwe National Statistics office classification was used to classify clusters into urban and rural as defined by population size and types of activity, age in five-year categories, sex (M/F), educational (no education, primary and secondary and above), employment status in the last 12 months (not employed, employed), wealth quintile (lowest & second, middle, forth & highest); (b) sexual behavioural factors that include currently in union (yes, no), age at first sexual intercourse (<16, > = 16), number of lifetime partners (1, 2, 3–4, 5–9, 10 or more), condom use at last sexual intercourse in past 12 months (yes, no); (c) biological factors that include sexual transmissible infections (STI) in past 12 months (had STI or STI symptoms, no STI or no symptoms) and male circumcision (yes, no).

Relative change in HIV prevalence was the main outcome variable and was calculated as the difference of prevalence in 2005/06 minus 2010/11 divided by prevalence in 2005/6 and presented as a percentage. In DHS data files the data is weighted to correct for over and under sampling in some regions hence the sample weights were used during the analysis. The use of sample weights during analysis ensures sample representativeness. The sample weight was an 8-digit variable with 6 implied decimal places. To use the sample weight we divided it by 1,000,000 before applying the weighting factor. All sample weights were normalized such that the weighted number of cases was identical to the unweighted number of cases when using the full dataset with no selection. The weight variable was used to weight all tabulations produced using the data file.

### Data Analysis

The change in HIV prevalence and risk factors were explored using the svy, mean command (for survey design analysis) in *Stata Corp Version 12*.*1* followed by a Lincom command (Linear combinations of estimators), which is equivalent to a t-test (t test for differences between two proportions). All continuous variables such as age, number of sexual partners were categorized for analysis as explained under variables. All analysis was two-tailed and the significance level was set at 0.05.

Logistic regression was performed for changes in HIV prevalence by regressing HIV results (positive/negative) with year variable indicator and including and controlling for all the socio-demographic, sexual risk behavior and biological factors. The co-efficient of year (reference category was year 2005/06) was used to obtain the adjusted odds ratio for change in HIV prevalence between the two surveys for the selected variable controlling for the other factors. This co-efficient was used to interpret the output of the logistic regression models fitted. Because male circumcision applies to males only, it was not used in the logistic regression model in order to have an accurate comparison of males and females. We analysed the men and women’s 2005/6 and 2010/11 ZDHS data sets separately.

### Ethical Consent

In the 2005/6 ZDHS standard verbal informed consent procedures were followed prior to blood sample collection. The sample was bar code labelled and personal identifiers were removed before linking with household behavioural data. The Medical Research Council of Zimbabwe (MRCZ) approved verbal consent without return of HIV results in an effort to increase participation rates. Participants were given coupons to access HIV testing in the national HIV testing and counselling program. In 2010/11 survey, participants gave informed written consent and consent forms were sent to the Zimbabwe National Statistical Agency (Zimstats) for storage. Both surveys were approved by ICF International review board and the US Office for the Protection from Research Risks (OPRR). This extended analysis was approved by the US Centers for Disease Control and Prevention (CDC), Center for Global Health (CGH).

## Results

The HIV prevalence and proportions of change in prevalence are summarized in Table 1, which shows major variations within gender, across the different variables. Nationally there was a significant decline in HIV prevalence among men of 15% (p = 0.011), and similarly women experienced a similar significant proportional decline of 16% (p = 0.008) between the 2005/6 and 2010/11 surveys. However, there were striking variations within gender for some variables. Among men, the change within province ranged from (-46%, p <0.001 Harare) to (+29%, p = 0.223 Bulawayo) and for women the range was (-34%, p = 0.055, Mashonaland Central) to (+8% p = 0.530 Bulawayo). While there was a higher decline in prevalence for men in urban (17%, p = 0.089) compared to rural (13%, p = 0.073) settings, the opposite was true among women with a higher and significant change in rural areas (19%, p = 0.002) and no significant change in urban areas 9% (p = 0.144). Younger women (15–34 years) experienced the higher declines ranging from (-35%, p<0.001, age 20–24 years) to (-18%, p = 0.012 age 30–34 years). The only increase in prevalence were observed among men 15–19 years (+10%, p = 0.679) and 45–49 years (+15%, p = 0.307) and among women 45–49 years (+25%, p = 0.111). The highest decline though not statistically significant was experienced in men (-32%, p = 0.351) and women (-24%, p = 0.277) without any educational background however significant declines were observed in both men (-16%, p = 0.012) and women (-19%, p<0.001) with above secondary education. For sexual risk behaviors there was a high proportional decline among both men (-23%, p<0.001) and women (-20%, p<0.001) who were in union and those who had reported their sexual debut at 16 years and older (men 23% p<0.001 and women (- 21% p<0.001). There were variations in decline of prevalence by number of sexual partners but increases were observed in women with 5 to 9 partners. Significant declines were observed for condom use in men (-33%, p<0.001) and women (-32%, p<0.001) while prevalence increased when condom use was not reported for men (29%, p = 0.03) and women (6%, p = 0.508).

**Table 1:** Change in HIV prevalence in Zimbabwe by sex between the 2005/6 and 2010/11 surveys by different socio-demographic characteristics (weighted).

		Men				Women			
Variable	Categories	ZDHS 2005/6	ZDHS 2010/11	Proportion of change	P value (diff) ^a^	ZDHS 2005/6	ZDHS 2010/11	Proportion of change	P value
Number tested		5,848	6,250		-	6,947	7,313		-
Total HIV (%)		14.5	12.3	-15%	0.008	21.1	17.7	-16%	0.011
Province	Manicaland	16.6	9.8	-41%	0.010	22.3	17.9	-20%	0.063
	Mashonaland Central	13.8	12.3	-11%	0.620	22.9	15.1	-34%	0.055
	Mashonaland East	14.4	13.2	-8%	0.654	21.3	17.8	-16%	0.161
	Mashonaland West	15.4	11.5	-25%	0.073	22.5	17.8	-21%	0.094
	Matebeleland North	14.4	16.1	12%	0.539	22.8	20.2	-11%	0.373
	Matebeleland South	15.6	19.3	24%	0.387	24.6	22.7	-8%	0.455
	Midlands	11.5	13.0	13%	0.526	20.1	17.4	-13%	0.289
	Masvingo	12.1	11.8	-2%	0.889	17.3	16.3	-6%	0.753
	Harare	17.3	9.3	-46%	<0.001	21.1	16.7	-21%	0.024
	Bulawayo	12.8	16.5	29%	0.223	19.6	21.1	8%	0.53
Location	Urban	15.7	13.1	-17%	0.089	21.6	19.6	-9%	0.144
	Rural	13.8	12.0	-13%	0.073	20.8	16.8	-19%	0.002
Age group	15–19	3.1	3.4	10%	0.679	6.2	4.2	-32%	0.031
	20–24	5.8	3.8	-34%	0.089	16.3	10.6	-35%	<0.001
	25–29	13.0	10.3	-21%	0.123	28.8	20.1	-30%	<0.001
	30–34	29.5	17.4	-41%	<0.001	35.5	29.0	-18%	0.012
	35–39	32.1	25.1	-22%	0.026	34.5	29.1	-16%	0.040
	40–44	32.9	26.2	-20%	0.078	25.7	25.7	0%	0.976
	45–49	26.0	29.9	15%	0.307	18.0	22.5	25%	0.111
Education	No education	23.4	15.8	-32%	0.351	20.0	15.2	-24%	0.277
	Primary	15.0	13.6	-9%	0.402	22.4	20.1	-10%	0.175
	Secondary and above	14.2	11.9	-16%	0.012	20.5	16.7	-19%	<0.001
Employment status in last 12 months	Not employed	8.3	9.1	10%	0.477	18.9	15.0	-21%	<0.001
	Employed	17.3	13.8	-20%	<0.001	24.0	21.4	-11%	0.063
Wealth quintile	Lowest & second	14.3	13.4	-6%	0.589	19.4	16.7	-14%	0.070
	Middle	12.2	12.0	-2%	0.910	22.7	19.9	-12%	0.201
	Fourth & Highest	15.5	11.5	-26%	<0.001	21.8	17.6	-19%	<0.001
Currently in union	No	4.3	4.0	-7%	0.628	8.4	7.6	-10%	0.406
	Yes	23.1	17.7	-23%	<0.001	20.2	16.1	-20%	<0.001
Age at first sexual intercourse	<16	16.3	14.5	-11%	0.502	27.0	23.6	-13%	0.109
	> = 16	19.5	15.0	-23%	<0.001	25.8	20.3	-21%	<0.001
Number of lifetime partners	1	6.5	4.0	-38%	0.063	18.1	12.1	-33%	<0.001
	2	14.8	12.3	-17%	0.298	37.1	32.2	-13%	0.024
	3 to 4	20.3	13.8	-32%	<0.001	42.1	40.6	-4%	0.619
	5 to 9	22.1	20.6	-7%	0.487	43.9	46.9	7%	0.656
	10+	31.2	27.2	-13%	0.228	76.2	40.6	-47%	0.002
Condom use at last sexual intercourse in past 12 months	Yes	21.3	14.3	-33%	<0.001	21.2	14.5	-32%	<0.001
	No	15.0	19.4	29%	0.032	39.1	41.4	6%	0.508
Sexually transmitted infections in past 12 months	Had STI or STI symptoms	14.0	12.0	-14%	0.013	19.9	17.0	-15%	0.002
	No STI, no symptoms	35.7	26.3	-26%	0.15	51.2	41.3	-19%	0.053
Male circumcision	No	14.2	12.2	-14%	0.017				
	Yes	16.6	14.1	-15%	0.313				

ZDHS, Zimbabwe Demographic and Health Survey; STI, Sexually transmitted infection

^a^ *p*-value significant if less than 0.05

In the logistic regression analysis (Table 2), the decline in HIV prevalence remained statistically significant for both men and women in three of ten provinces, i.e Manicaland, Mashonaland East and Harare and for women only in Mashonaland Central. The change in prevalence in rural and urban location which had been masked in the univariate analysis became significant in the regression analysis. The decline in prevalence among young women (15–29 years) and men in the age range 30-44years remained statistically significant. There were significant declines among men and women regardless of educational level except in men with primary and women with no education. Similarly, there were significant declines irrespective of employment or wealth status with the exception of men in the lowest and second quintiles.

**Table 2:** Logistic regression of the factors associated with change in HIV prevalence among adults aged 15–49 years who were tested for HIV, ZDHS 2005/6 and 2010/11.

		Men				Women			
Variable	Categories	AOR	P-value ^a^	95% C.I		AOR	P-value	95% C.I	
National		0.71	<0.001	0.60	0.84	0.66	<0.001	0.58	0.75
Province	Manicaland	0.43	<0.001	0.26	0.72	0.66	0.02	0.45	0.94
	Mashonaland Central	0.66	0.15	0.38	1.17	0.39	0.01	0.21	0.75
	Mashonaland East	0.58	0.03	0.34	0.95	0.44	<0.001	0.27	0.72
	Mashonaland West	0.76	0.27	0.47	1.24	0.67	0.06	0.44	1.01
	Matebeleland North	1.08	0.74	0.66	1.78	0.79	0.27	0.51	1.21
	Matebeleland South	1.07	0.84	0.52	2.21	0.81	0.28	0.55	1.19
	Midlands	1.03	0.90	0.63	1.69	0.75	0.16	0.50	1.12
	Masvingo	0.68	0.11	0.43	1.09	0.73	0.10	0.50	1.06
	Harare	0.45	<0.001	0.30	0.68	0.57	<0.001	0.41	0.80
	Bulawayo	-	-	-	-	0.82	0.33	0.55	1.22
Residence	Urban	0.73	0.02	0.55	0.96	0.75	0.00	0.61	0.90
	Rural	0.68	<0.001	0.55	0.86	0.61	<0.001	0.51	0.72
Age	15–19	0.74	0.59	0.26	2.16	0.49	0.01	0.29	0.84
	20–24	0.59	0.08	0.33	1.06	0.60	<0.001	0.46	0.78
	25–29	0.75	0.11	0.52	1.07	0.52	<0.001	0.41	0.68
	30–34	0.52	<0.001	0.38	0.72	0.65	0.001	0.49	0.86
	35–39	0.66	0.01	0.48	0.91	0.76	0.07	0.57	1.02
	40–44	0.66	0.04	0.45	0.98	0.86	0.54	0.55	1.37
	45–49	1.08	0.75	0.66	1.77	1.57	0.14	0.87	2.83
Education	No education	0.01	0.02	0.00	0.49	1.08	0.85	0.47	2.48
	Primary	0.78	0.17	0.55	1.11	0.74	<0.001	0.60	0.91
	Secondary and above	0.66	<0.001	0.55	0.79	0.60	<0.001	0.51	0.70
Currently working	Not employed	0.54	<0.001	0.37	0.81	0.64	<0.001	0.54	0.76
	Employed	0.72	<0.001	0.60	0.87	0.68	<0.001	0.55	0.83
Wealth quintile	Lowest & second	0.78	0.14	0.56	1.08	0.66	<0.001	0.54	0.82
	Middle	0.60	0.01	0.42	0.87	0.53	<0.001	0.40	0.71
	Fourth & Highest	0.69	<0.001	0.55	0.86	0.68	<0.001	0.57	0.82
Currently in union	No	0.67	0.15	0.39	1.16	0.56	0.01	0.36	0.88
	Yes	0.66	<0.001	0.54	0.80	0.65	<0.001	0.56	0.75
Age at first sexual intercourse	<16	0.75	0.25	0.46	1.23	0.77	0.09	0.57	1.04
	> = 16	0.67	<0.001	0.56	0.80	0.63	<0.001	0.55	0.73
Number of lifetime partners	1	0.56	0.03	0.33	0.95	0.60	<0.001	0.50	0.72
	2	0.68	0.07	0.45	1.03	0.71	<0.001	0.56	0.89
	3+	0.55	<0.001	0.41	0.75	0.73	0.04	0.54	0.99
Condom use at last sexual intercourse in past 12 months	Yes	0.59	<0.001	0.48	0.72	0.60	<0.001	0.52	0.70
	No	1.12	0.48	0.81	1.56	0.89	0.49	0.65	1.23
Sexually transmitted infections in past 12 months	Had STI or STI symptoms	0.70	<0.001	0.59	0.83	0.66	<0.001	0.57	0.76
	No STI, no symptoms	0.46	0.04	0.21	0.97	0.66	0.11	0.39	1.11
Male circumcision	No	0.71	<0.001	0.59	0.85				
	Yes	0.59	0.02	0.38	0.92				

AOR, Adjusted Odds Ratio; STI, Sexually transmitted infection

^a^ *p*-value significant if less than 0.05

Analysis of sexual risk behaviours showed non-significant declines only in men who were not currently in union. Regardless of the number of sexual partners, there was a significant decline in both men and women. Other significant determinants included sexual debut above 16 years and condom use in both men and women. Regardless of reported STI history, there was a decline among men and women, although these were significant among those with previous STI exposure.

## Discussion

Our study shows that while nationally the declines among women and men appeared similar, there are striking variations in the change in prevalence across the different provinces, urban and rural location, age and sexual risk behavior within each gender. The similarities at national level suggest that interventions may have had a similar effect on both men and women. These variations support studies that show that national trends mask the real differences. [2,5]

The high proportional decline in HIV prevalence observed among men of (-) 46% in Harareand women of -34% in Mashonaland Central compared to an increase in prevalence of (+) 24% among men in Matebeleland North and (+) 8% among women in Bulawayo; suggest that the epidemic is heterogeneous and that there are significant differences that require further examination to determine reasons for these variations by gender and provincial location. Possible explanations include the fact that areas which experienced an increase in prevalence are all situated in a geographic location that is close to a border or are situated around a highway from a border. This is the case for Matebeland North and South, Bulawayo and Midlands where increases maybe related to high internal and inter-country migration due to economic reasons.[15] Such variations warrant a data driven focused approach in HIV programming. The pronounced decline in prevalence among young women in age groups 15–39 years may suggest reduced HIV incidence in a group that has been disproportionately known to be at a higher risk of HIV infection. [3] A decrease in this age-group is also suggestive of a decline in HIV incidence^5^. This study highlighted increases in HIV prevalence among older men and women this maybe a function of reduced mortality attributable to survival on antiretroviral therapy. A review of mortality records by Dlodlo et al (2011) suggested that the declining trend in mortality in the two major cities could be associated with treatment scale up. [16]

Our study indicates that high education (secondary and above) for both males and females is associated with significant decline in HIV prevalence, while female with no education remain vulnerable to HIV infection with an insignificant decline recorded between the two surveys (p>0.05). Mbanga et al observed a shift in the HIV epidemic from educated to the uneducated.[7] Given the continued focus on education in Zimbabwe and the high literacy rate has a potential in contributing f to further reductions of HIV prevalence in Zimbabwe. This analysis supports theories that adoption of positive sexual risk behaviours in Zimbabwe such as increase in age of sexual debut and condom use can explain the decline in HIV prevalence.[17,18] Marital union and monogamy are positively associated with the decline for both men and women. Although numbers tend to be smaller, the numerically high prevalence among men and women reporting more than one sexual partner are a cause of concern and requires follow-up research using proven methods of collecting sensitive information such as the Audio Computer- Assisted Self–Interview (ACASI) supported by collection of HIV blood for biomarkers.[19] These findings underscore the need for interventions to increase age of sexual debut, reduction in sexual partners and promote condom use.

The finding that there is a similar proportional decline in HIV prevalence among men who had been circumcised compared to those who had not been circumcised cannot be interpreted to infer causality, because of the small numbers involved. Clinical trials have demonstrated a nearly 60% reduction in the risk of HIV transmission among men aged between 15 and 49 years who became circumcised. [20] The finding in our study needs to be interpreted with caution given that this information is self- reported and that medical male circumcision had not been implemented during this period.

Finally, the HIV epidemic is currently in a pattern of decline in many areas of Zimbabwe. A multi-centre study of HIV epidemics in Sub-Saharan Africa attributed such a change to the natural progression of the epidemic, a decrease in risky practices, and increased access to treatment.[21] Regardless of direction of change in prevalence, this analysis highlights the importance of a disaggregated analysis in order to focus interventions.

### Limitations

One of the limitations of our study is that it focused on the changes in HIV prevalence alone. This was constrained by the design of the Demography and Health Surveys which are cross-sectional in nature and provides limited opportunities to assess changes in HIV incidences. [13,14] This information is critical in the broad strategies of fighting the HIV pandemic in Zimbabwe. Future research efforts should be directed to determine the feasibility of calculating general population HIV incidences based on ZDHS data and other reliable population based studies. However, this analysis provides results that are of essence to other HIV programming activities such as PMTCT, ANC, youth HIV programs and blood safety program [22] as these can identify changes noteworthy to incorporate into their programming activities. This analysis is not able to determine the actual contribution of mortality and migration on the observed declines in HIV prevalence hence should be interpreted with caution.

## Conclusions

This analysis shows that while Zimbabwe has reported a significant decline among both men and women, there are important differentials that suggest that the epidemic in Zimbabwe is heterogeneous and warrants closer examination of these variations. The changes in HIV prevalence were more pronounced in some provincial regions, among young women and is associated with adoption of safer sexual behaviours.

### Disclaimer

The views expressed are those of the authors and do not necessarily reflect the views of USAID, CDC, the United States Government or the European Union. ICF international, Calverton, Maryland, USA, provided technical assistance for the analysis and preparation of the paper.
